# Identifying factors associated with user retention and outcomes of a digital intervention for substance use disorder: a retrospective analysis of real-world data

**DOI:** 10.1093/jamiaopen/ooad072

**Published:** 2023-09-02

**Authors:** Franziska Günther, David Wong, Sarah Elison-Davies, Christopher Yau

**Affiliations:** Division of Informatics, Imaging & Data Sciences, School of Health Sciences, University of Manchester, Manchester M13 9GB, United Kingdom; Division of Informatics, Imaging & Data Sciences, School of Health Sciences, University of Manchester, Manchester M13 9GB, United Kingdom; TELUS Health, Manchester M15 6SE, United Kingdom; Nuffield Department of Women’s & Reproductive Health, University of Oxford, Oxford OX3 9DU, United Kingdom; Health Data Research UK, London NW1 2BE, United Kingdom

**Keywords:** digital health intervention, secondary use, substance use disorder, real-world uptake, real-world data exploration

## Abstract

**Objectives:**

Successful delivery of digital health interventions is affected by multiple real-world factors. These factors may be identified in routinely collected, ecologically valid data from these interventions. We propose ideas for exploring these data, focusing on interventions targeting complex, comorbid conditions.

**Materials and Methods:**

This study retrospectively explores pre-post data collected between 2016 and 2019 from users of digital cognitive behavioral therapy (CBT)—containing psychoeducation and practical exercises—for substance use disorder (SUD) at UK addiction services. To identify factors associated with heterogenous user responses to the technology, we employed multivariable and multivariate regressions and random forest models of user-reported questionnaire data.

**Results:**

The dataset contained information from 14 078 individuals of which 12 529 reported complete data at baseline and 2925 did so again after engagement with the CBT. Ninety-three percent screened positive for dependence on 1 of 43 substances at baseline, and 73% screened positive for anxiety or depression. Despite pre-post improvements independent of user sociodemographics, women reported more frequent and persistent symptoms of SUD, anxiety, and depression. Retention—minimum 2 use events recorded—was associated more with deployment environment than user characteristics. Prediction accuracy of post-engagement outcomes was acceptable (Area Under Curve [AUC]: 0.74–0.79), depending non-trivially on user characteristics.

**Discussion:**

Traditionally, performance of digital health interventions is determined in controlled trials. Our analysis showcases multivariate models with which real-world data from these interventions can be explored and sources of user heterogeneity in retention and symptom reduction uncovered.

**Conclusion:**

Real-world data from digital health interventions contain information on natural user-technology interactions which could enrich results from controlled trials.

## Background and significance

Substance use disorders (SUDs) are widely recognized as a major contributor to global disease burden.^1^ The impact of SUDs is intensified by consistently low SUD treatment rates,^2^ which result from a variety of factors. Among these are variable access to evidence-based treatment due to financial barriers,^3^ place of residence,^4^ treatment service workforce capacity,^5^ and recently, the circumstances of a global pandemic.^6^ Other factors are linked to the unavailability of treatment services at times of increased risk of substance use or relapse, for example, out of treatment service operating hours^7^ or during periods of transition between treatment settings, for example, when re-entering the community after a custodial sentence.^8^ Further, fear of stigmatization^9^ and an unstructured lifestyle centering around substance provision and consumption may prevent attendance of treatment appointments.

In contrast to traditional face-to-face delivery, digital delivery promises to widen access to SUD treatment by being anonymous, scalable, and accessible anywhere and anytime. So-called digital interventions (DIs) make therapeutic content available on the internet, via telephone or video chat, or within a smartphone app. DIs for mental and physical health may accumulate large quantities of individual-level usage data, often in order to personalize content, but these data can also provide information on real-time user experience and recovery progression.^10^ Further, the ecological validity of these data contrasts with that collected in highly controlled studies, such as randomized control trials (RCTs), in which the natural interaction of a user with a DI may be perturbed by study participant selection, monitoring, and incentives.^11^^,^^12^ As sustainable uptake of many digital health interventions remains low despite rigorous study of their effectiveness,^13^ post-deployment exploration of ecologically valid data may produce novel insights into real-world efficacy of the DI.

Little research in this area exists to date. Typical research goals are the identification of determinants of DI success, including sociodemographic user characteristics, baseline case severity, or engagement frequency. Success has been defined in terms of diagnostic outcomes, program completion, or retention. Chien et al.^14^ used a probabilistic latent variable model to identify user subgroups within longitudinal engagement data from a DI for anxiety and depression. Bell et al.^15^ employed k-modes clustering with a similar agenda and describe the role of notifications for daily engagement patterns of users of a DI for the reduction of harmful and hazardous alcohol use in the general population. Titov et al.^16^ reported yearly trends in user characteristics and outcomes for a national DI for anxiety and depression. Ramos et al.^17^ predicted goal attainment and completion of a linear DI program for alcohol, marijuana, and tobacco use with a random forest, using engagement data from the first 3 days of use.

More research into the impact of user heterogeneity on intervention outcomes, however, is warranted, especially analyses of data from interventions targeting individuals with complex and transdiagnostic symptom profiles common in mental health contexts.

## Objectives

In this study, we conduct a retrospective analysis of real-world data from a DI to understand the factors associated with its deployed performance. We use the example of “Breaking Free Online” (BFO), a cognitive behavioral therapy (CBT) DI widely established in SUD treatment services in the United Kingdom. Our analyses focus on static user characteristics as a source of heterogenous responses to the DI, and the prediction of post-engagement symptomatology.

## Materials and methods

This study is a retrospective, exploratory, one-group pretest–post-test study of individuals accessing 477 SUD treatment services in England, Scotland, and Wales between January 4, 2016 and December 6, 2019, with each agreeing to create a BFO account.

BFO is a tailorable, CBT-based DI program offered by commissioning SUD treatment services in the community or in correctional environments.^18^^,^^19^ Unlimited access to clinical content on personally or service owned devices is provided free of charge. Users are required to complete an integrated digital questionnaire-based assessment of their recovery progression and sociodemographic background before first access and at least fortnightly after that. Care was taken to ensure assistance with the program is available to users at participating services, and to closely integrate the program with face-to-face clinical practice.^20^ Further detail of the program, for example, on client onboarding and program content is presented in Supplementary Material.

To tailor BFO content to an individual user’s recovery progression and challenges, users are required to provide consent for the collection, storage, and use of their non-identifiable data. Specific consent to use of data for research is not required for access to BFO. The program allows users to download any data accumulated through their engagement with the program, and to launch the process of purging their data if they wish to no longer access BFO.

This study analyzes user self-reported data from the first- and, if available, last-contact assessment. In the following, assessment times will be referred to as “pre-engagement” and “post-engagement”. This relates to our decision to consider those dropouts who have not completed the assessment at least twice and hence did not engage with the program in the recommended way. Those who did not dropout are subsequently referred to as “retained.”

Ethical approval for collection, storage, and use of data accumulating from routine use of BFO by clients in participating treatment services, was obtained from an NHS Research Ethics Committee (London—South East, May 22, 2017, reference 12/LO/0287).

### Data description

The BFO assessment combines a variety of items associated with validated and standardized psychometric scales, the Severity of Dependence Scale (SDS), the Patient Health Questionnaire 4 (PHQ-4), the World Health Organization Quality of Life measure (WHOQOL-BREF), and the Recovery Progression Measure (RPM), and additionally collects data on the substance-using days in the preceding week. In the following, we will refer to “recovery progression” as the construct measured within the BFO assessment rather than the RPM.

The SDS^21^ is a 5-item scale from 0 to 3 measuring the degree of dependence on a substance. If the total of the answers on these items ≥3, an individual is considered dependent. Users addressing both alcohol and drug use with BFO are presented with the SDS twice to assess their dependence on each substance. Pre-engagement, users are presented with an additional item on the impact their target substances has on them.

The PHQ-4^22^ is a 4-item screener for depression and anxiety on a scale from 0 to 3. The total of the answers on the first, or last 2 items, meeting a cutoff of 3 is considered an indicator of anxiety or depression, respectively. The PHQ-4 is presented together with an item from the RPM on the impact of emotions. Pre-engagement, an additional item on negative self-perception is presented to users alongside the PHQ-4. Additional items are presented to align with the RPM subscale format, that is, 5 items on a specific aspect of functioning plus one item on this aspect’s impact.

Five items (number 1, 2, 17, 18, 20) from the WHOQOL-BREF^23^ are presented alongside 2 items on overall satisfaction with life and ability to cope with life’s difficulties.

From the RPM,^24^ a 36-item scale measuring biopsychosocial functioning during SUD recovery, subscales, “unhelpful behaviours”, “lifestyle”, “physical sensations”, “difficult situations”, and “negative thoughts” are used. Post-engagement, a 6-item screening version of the RPM^25^ is used.

### Statistical analysis

We included individuals who completed the pre-engagement assessment, defined as answering at least one item associated with a scale, and sub-scale in the case of the RPM.

Sociodemographic characteristics of the population for which BFO was an attractive option when accessing an SUD treatment service, and their time of engagement with BFO in weeks were analyzed descriptively.

Several regression models were used to understand the association of participant characteristics with retention and post-engagement outcomes. Among these was a multivariate, multivariable ordinal logistic regression model inspired by^26^ which is faithful to the ordinal measurement level of data self-reported on questionnaires, and the high correlations which often exist between questionnaire items (see Figure S1). We illustrate the merit of this methodological approach in describing the association of participant characteristics and post-engagement outcomes through the example characteristic of gender, as the role of gender in SUD treatment continues to be specified in the scientific literature.^27^ However, the approach can be adapted to explore the interplay of other participant characteristics and DI retention and outcomes. To avoid bias of estimates of the effect of gender, as well as of statements about transgender individuals with SUD, we did not include transgender individuals in our analysis, pertaining to their small proportion of the sample (0.9%). Details of statistical models can be found in Table 1. An example for one of our multivariate, multivariable models would take age, gender, ethnicity, service, target substance, assessment time, and the interaction between gender and assessment time as inputs to model answering patterns on all SDS items.

**Table 1.** Models used to understand associations of participant characteristics with retention and outcomes.

Regression model	Outcome	Inputs	Example
Univariate multivariable binary logistic	Meeting of cutoff for anxiety, depression, or substance dependence	Age, gender, ethnicity, service, target substance, retention,^b^ assessment time,^c^ gender * assessment time^c^	“pre-engagement meeting of cutoff for anxiety ∼ age + gender + ethnicity + service + target substance + retention”
Univariate multivariable ordinal logistic	Number of substance—using days per week		“number of alcohol- using days per week ∼ age + gender + ethnicity + service + target substance + assessment time + gender * assessment time”
Multivariate multivariable ordinal logistic^a^	Items originating from or presented alongside with SDS, PHQ-4, WHOQOL-BREF, RPM subscale,^b^ Rapid RPM^c^		“SDS ∼ age + gender + ethnicity + service + target substance + assessment time + gender * assessment time”
Univariate multivariable binary logistic	Retention		“retention ∼ age + gender + ethnicity + service + target substance”

a To ensure identifiability, we followed recommendations in Ref.^26^ to fix intercept parameters to zero and error variance to unity for all models.

b Included in model of pre-engagement assessment.

c Included in model of pre- and post-engagement assessment.

Reliable recovery was defined as the proportion of participants meeting the cutoff for substance dependence, depression, or anxiety pre-engagement, but not post-engagement; or abstinence in terms of zero substance-using days in the preceding week post-engagement, but not pre-engagement. Reliable deterioration was defined as the opposite.

We predicted participant retention and post-engagement outcomes with random forests, using the full questionnaire and sociodemographic data available from the pre-engagement assessment. Predictions were made in 10 repetitions of 10-fold cross-validation. Predictor importance was assessed by computing the mean decrease in accuracy at random permutations of a predictor in each fold. Resulting ranks were aggregated for each predictor across folds and outcomes predicted by the same set of predictors. As these account for correlations between predictors, accumulated local effects^28^ were used to visualize effects of pre-engagement answers on the 10 most important drivers of prediction, using post-engagement anxiety as an example outcome.

To better understand the characteristics of users for which our prediction model fails, we employed binary logistic regression models to identify associations of repeated participant misclassification (model output) with participant sociodemographic characteristics and their meeting of cutoffs for substance dependence, depression, or anxiety (model input). We defined repeated misclassification as incorrect prediction in 100% of cases across cross-validation folds. Details of prediction models can be found in Supplementary Material.

We encounter missing data on some variables which we planned to integrate into our data models. Missingness in our case is a result of technical failure to record participant-input answers or to remind participants of missing input. The strategies for dealing with missing data are illustrated in Table 2. As our complete case analyses never result in the exclusion of more than 5% of cases, we deem our decision for them appropriate (multivariate imputation will likely not influence results). When we exclude the weekly number of substance using days from predictor sets or decide not to use the variable as an outcome in the model, we do this knowing that meeting the cutoff for clinical SUD gives us a reasonably good proxy for an individual’s substance dependence.

**Table 2.** Missing values on variables and strategies of dealing with them.

Variable name	Assessment time	Number of missing data	Strategy
Impact of alcohol	Pre-engagement	1^a^	Tolerance^a^
Alcohol using days	Pre-engagement	5^a^, 1^a^^,^^b^	Complete case analysis^a^, exclusion^b^
Alcohol using days	Post-engagement	3^a^^,^^c^	Complete case analysis^a^, exclusion^c^
Drug using days	Pre-engagement	10^a^, 0^b^	Complete case analysis^a^, exclusion^b^
Drug using days	Post-engagement	0^c^	Exclusion^c^
Coping with life’s difficulties	Post-engagement	495^a^^,^^c^	Tolerance^a^, exclusion^c^

a Outcome in ordinal regression model.

b Predictor in random forest model.

c Outcome in random forest model.

*Note*: Model tolerance of substantial amounts of missing data can be assumed for the multivariate, multivariable ordinal regression models by Ref.^26^ Two numbers of missing data for a variable indicate different numbers of missing data for the datasets with and without dropouts. Complete variables may be excluded from models because their equivalents for another target substance are excluded (see Drug using days).

## Results

### Participant description

Across the study period, 14 078 users of addiction services in England, Scotland, and Wales created a BFO account. From these registrations, 12 529 (90%) initially completed BFO’s assessment battery which is required to access clinical content and were eligible for statistical analysis (see Figure S2). This cohort was characterized by a mean age of 40.28 years (SD: 11.67, range: 18–99), and a proportion of 43% (5346) identifying as women. The proportion of participants identifying as White was 93% (11 703), 2% (243) identified as Asian/Asian British, 2% (214) identified as Black/Black British, 2% (299) as having a mixed ethnic background, and 1% (70) as having another ethnicity than the ones specified. A total of 93% (11 606) of participants accessed BFO through community services, while 7% (923) obtained access through correctional addiction services. Users sought help for their use of alcohol (57%, 7090), drugs (22%, 2708), and for the use of more than one substance (22%, 2731). Within the cohort, 66% (8235) identified alcohol as their most problematic substance, 9% (1132) named cocaine, 8% (960) heroin, 6% (805) marijuana, and 5% (567) crack.

The sociodemographic characteristics of and substances used by the cohort reflect those of the population seeking help for their substance use in England and Wales, which 99% of BFO users in this study participate from, except that BFO attracts more women and more individuals seeking help for alcohol use.^29^^,^^30^

### User characteristics associated with participant retention

Outcomes of DIs have to be evaluated in the light of how many users these interventions were able to retain. Retention can be low, especially when they cater to individuals whose condition involves strong cognitive pre-occupations and high risk of relapse like SUD, which can interfere with regular engagement. We found BFO retained 23% (2925) of participants for an average of 9 weeks (SD: 17 weeks, range: 0 days–142 weeks).

**Figure 1.**
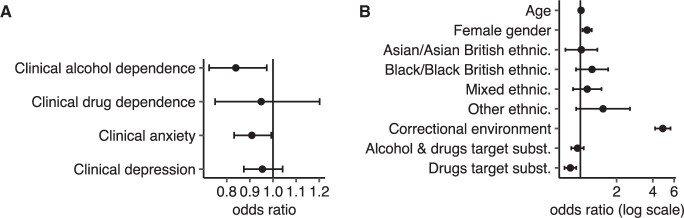
Association of participant retention with pre-engagement recovery progression and sociodemographic characteristics. Association analysis showing (A) odds ratios of participant retention against 4 clinical outcome measures; (B) odds ratios of association with sociodemographic membership. Reference groups are male gender, White ethnicity, community environment, and alcohol as a target substance. ORs are shown with their 95% confidence intervals.

We suspected participant dropout to be non-random and partly driven by specific, quantifiable differences between participants at baseline and found statistically significant associations between dropout and answering patterns on 13 out of a total of 57 items measuring participants’ recovery progression at baseline (see Figure S4). Figure 1A shows that meeting the cutoff for clinical anxiety or alcohol dependence (OR${}_{\text{anxiety}}$ = 0.91, *P* = .033, OR${}_{\text{alcohol dependence}}$ = 0.84, *P* = .020) was associated with dropout. While male gender (OR = 1.14, *P* = .004) and intending to address drug use with BFO (OR = 0.82, *P* = 7.29 ×10^−4^) were also associated with participant dropout, accessing BFO from a community SUD service (OR = 4.83, *P* = 9.22 ×10^−98^) showed the strongest association (Figure 1B).

Retrospective analysis of BFO data has therefore identified that participant dropout is highly linked with certain deployment environments, with certain user characteristics and baseline symptoms playing a smaller or no statistically significant role.

### Association of post-engagement outcomes with gender

We next investigated whether the post-engagement outcomes of the DI were associated with self-reported user characteristics. We examined aggregated and item-level outcomes and used gender as an example of a particular characteristic of interest both as a main effect but also its statistical interaction with pre- or post-engagement status.

**Figure 2.**
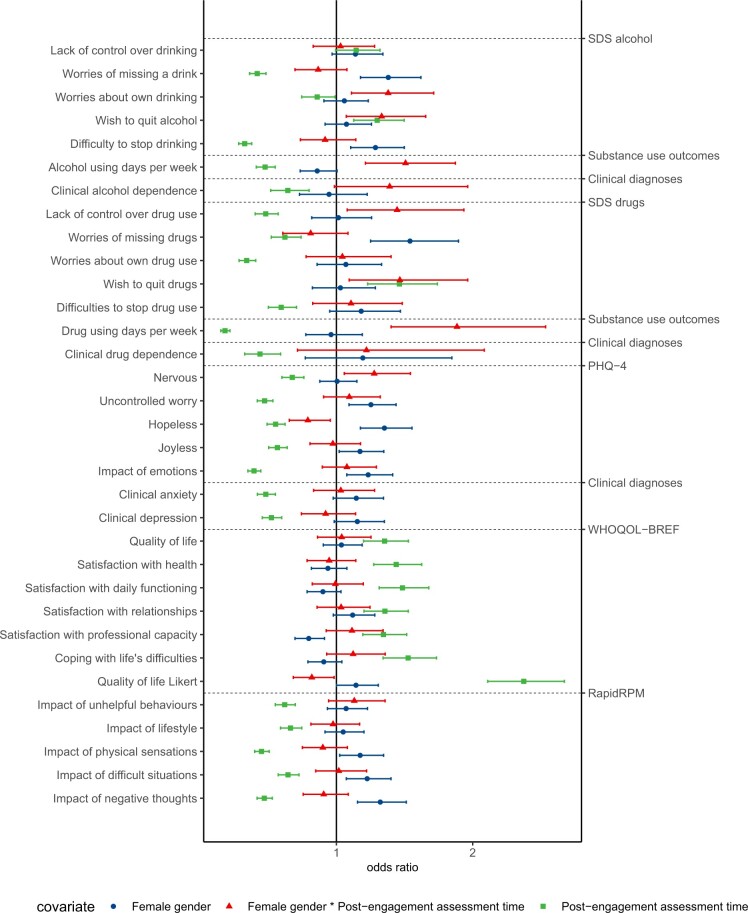
Associations of participant recovery progression measured on item and aggregate levels with assessment time pre- and post-BFO engagement, and gender. Odds ratios (reference groups: pre-engagement, male, and pre-engagement male) are shown with their 95% confidence intervals. The PHQ-4 and the WHOQOL-BREF are shown with affiliated items.

**Figure 3.**
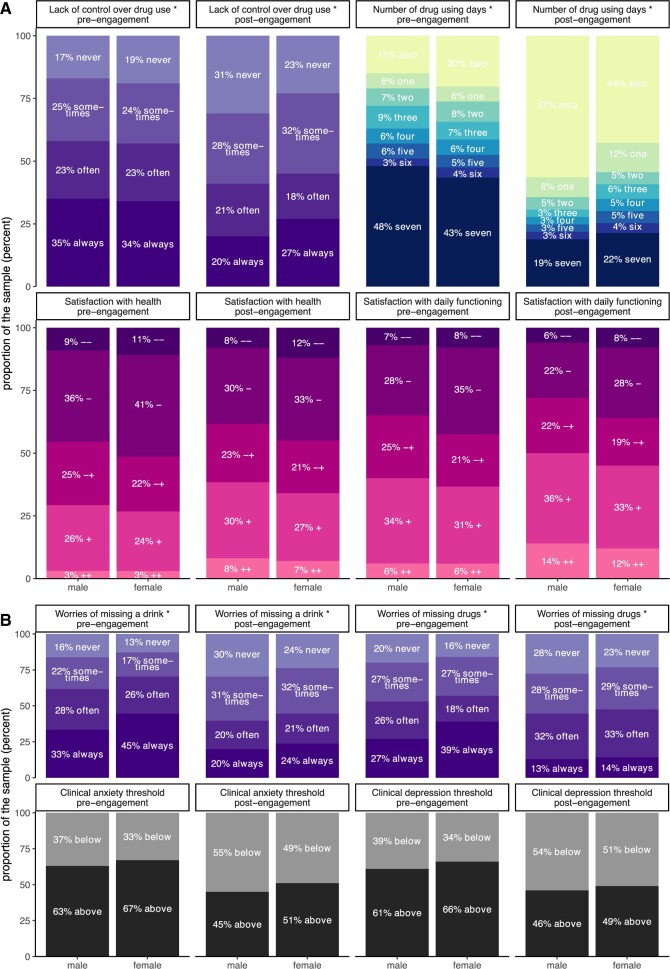
Subgroup questionnaire response illustration. Stacked bar plots showing pre- and post-BFO engagement differences in responses between men and women on a selection of questionnaire items and in terms of exceeding the cutoff for anxiety and depression. Each color block reflects a different categorical level for the outcome displayed. An asterisk indicates statistical significance of the interaction between assessment time and gender for (A), and statistical significance of the difference between men and women for (B), suggested by regression models.

Specifically, we found significant associations between female gender and increased anxiety at the prospect of missing an opportunity to use a drug (OR = 1.54, *P* = 7.81 ×10^−5^) or alcohol (OR = 1.38, *P* = 4.66 ×10^−5^), difficulty of stopping drinking (OR = 1.29, *P* = .001), frequency of uncontrolled worry (OR = 1.25, *P* = .001), hopelessness (OR = 1.35, *P* = 2.28 ×10^−5^), joylessness (OR = 1.17, *P* = .025), impact of physical sensations (OR = 1.17, *P* = .021), negative thoughts (OR = 1.32, *P* = 5.25 ×10^−5^), difficult situations (OR = 1.23, *P* = .003), and emotions (OR = 1.23, *P* = .003), and decreased satisfaction with professional capacity (OR = 0.80, *P* = .001), independent of BFO engagement (see Figures 2 and 3). Female gender was also significantly associated with slightly increased quality of life (OR = 1.14, *P* = .049).

Significant interaction effects between assessment time and gender were found on items on the number of alcohol- and drug-using days per week (OR${}_{\text{alcohol}}$ = 1.51, *P* = .0002, OR${}_{\text{drugs}}$ = 1.88, *P* = 2.77 ×10^−5^, see Figure S5), worries about alcohol use (OR = 1.38, *P* = .004), wish to stop using substances (OR${}_{\text{alcohol}}$ = 1.33, *P* = .010, OR${}_{\text{drugs}}$ = 1.47, *P* = .010), experienced lack of control over drug use (OR = 1.45, *P* = .013), quality of life (OR = 0.82, *P* = .032), nervousness (OR = 1.28, *P* = .011), and hopelessness (OR = 0.79, *P* = .015), suggesting slower reduction in recovery progression for women while engaged with BFO.

Odds ratios, for example, for weekly drug-using days can be interpreted as such: While across assessment times women do not differ from men regarding their odds of using drugs on more rather than less days, odds are decreased post- compared to pre-engagement regardless of gender. The impact of gender depends on the assessment time such that the odds of using drugs on more days is increased post-, but not pre-engagement for women compared to men.

In conclusion, we found that female substance users reported more severe psychiatric symptoms which largely do not reduce as readily as those of men following use of BFO. Similar analyses based on ethnicity were not possible with this data due to the low numbers of non-White participants.

### Outcome prediction

**Figure 4.**
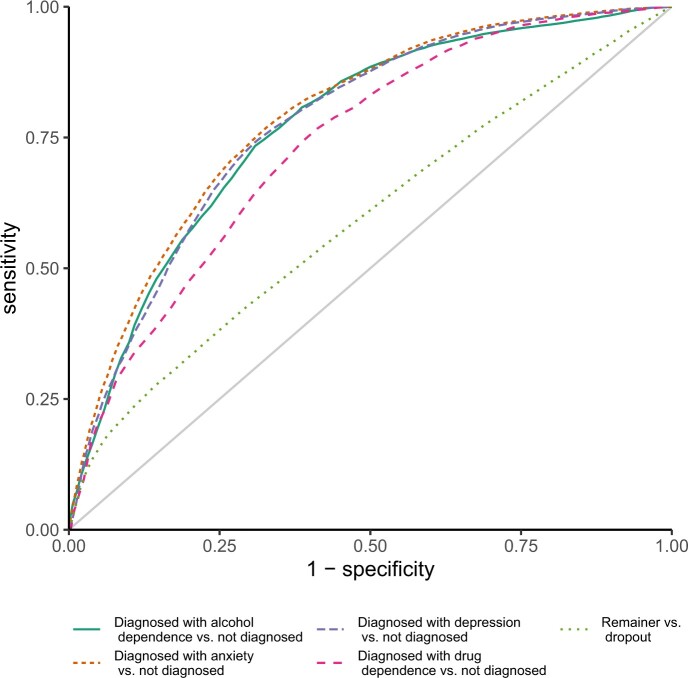
Averaged receiver operating characteristic curves for binary outcomes. The curve shows the averaged prognostic performance of random forests in 100 validation datasets. AUC${}_{\text{retention}}$ = 0.59, AUC${}_{\text{anxiety}}$ = 0.78, AUC${}_{\text{depression}}$ = 0.79, AUC${}_{\text{alcohol dependence}}$ = 0.77, AUC${}_{\text{drug dependence}}$ = 0.74.

Given these insights, we next set out to evaluate the possibility of building a risk prediction model to stratify users from their pre-engagement response profile on the initial assessment battery. Therefore, we examined whether a participant’s retention and outcomes can be predicted by training a random forest-based prediction model. Figure 4 shows acceptable predictive capability for a number of post-engagement clinical outcomes (area under the receiver operating characteristic curve, abbreviated as AUC = 0.74–0.79), but poor predictive capability for retention (AUC = 0.59). This indicates that outcome heterogeneity of the DI can be partially explained by initial user characteristics (see Figure S6 for performance on individual level items).

**Figure 5.**
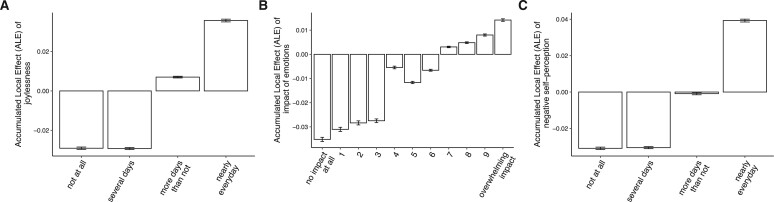
Accumulated local effects of 3 features on the prediction of anxiety. The predicted probability of post-engagement anxiety increased with pre-engagement levels of (A) joylessness, (B) impact of emotions, and (C) negative self-perception.

We explored further by using Accumulated Local Effects plots to examine the variables driving the predictions of functioning- and substance-related outcomes (Table 3). Examples of effects of answers on these drivers are presented in Figure 5 and Figure S7. These showed that post-engagement anxiety was increased against an initial backdrop of joylessness, a greater impact of emotions, and negative self-perception prior to BFO engagement.

**Table 3.** Drivers of prediction of post-engagement outcomes with random forests, ranked by importance.

Rank	Predictor of functioning	Predictor of alcohol dependence severity	Predictor of drug dependence severity
1	Joyless	Lack of control over drinking	Difficulty to stop drug use
2	Impact of emotions	Worries of missing a drink	Worries about own drug use
3	Negative self-perception	Worries about own drinking	Lack of control over drug use
4	Coping with life’s difficulties	Difficulty to stop drinking	Wish to quit drugs
5	Quality of life Likert	Clinical alcohol dependence	Impact of drugs
6	Impact of unhelpful behaviors	Wish to quit alcohol	Negative self-perception
7	Satisfaction with daily functioning	Impact of alcohol	Joyless
8	Impact of negative thoughts	Impact of physical sensations	Impact of negative thoughts
9	Nervous	Satisfaction with health	Impact of physical sensations
10	Impact of physical sensations	Negative self-perception	Coping with life’s difficulties

Ranks are aggregated across functioning, alcohol dependence, and drug dependence related outcomes, respectively.

**Figure 6.**
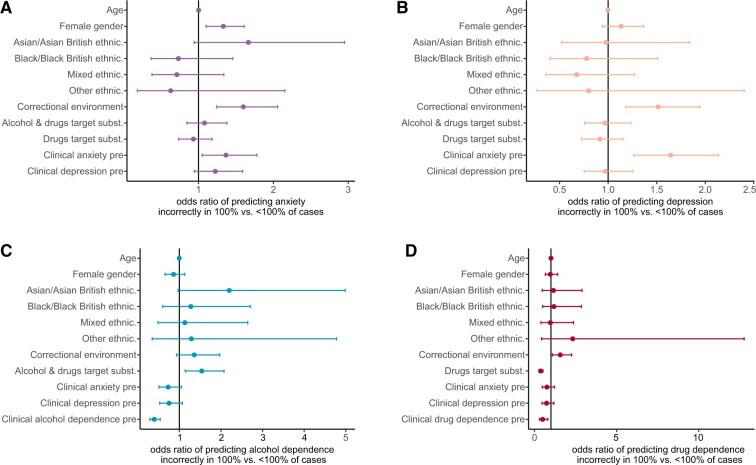
Associations of repeated misclassification post-engagement with pre-engagement participant characteristics. Reference groups are male gender, white ethnicity, community environment, alcohol as a target substance for associations with the misclassification of post-engagement (A) anxiety, (B) depression, and (C) alcohol dependence, and alcohol and drugs as target substances for associations with the misclassification of post-engagement (D) drug dependence. ORs are shown with their 95% confidence intervals.

We next decided to examine associations between pre-engagement characteristics of participants and prediction failure, which are detailed in Figure 6. We found significant associations between accessing BFO from a correctional SUD service and repeatedly wrong predictions of anxiety, depression, and drug dependence (OR${}_{\text{anxiety}}$ = 1.60, *P* = .0003, OR${}_{\text{depression}}$ = 1.52, *P* = .001, OR${}_{\text{drug dependence}}$ = 1.57, *P* = .016). Individuals in correctional, compared to those in community services have hence increased odds of being repeatedly misclassified by our models. Associations between repeatedly wrong predictions of alcohol and drug dependence and the intention to address both alcohol and drug use with BFO (OR${}_{\text{alcohol dependence}}$ = 1.54, *P* = .005, OR${}_{\text{drug dependence}}$ = 0.37, *P* = 6.59 ×10^−9^) as well as undershooting the cutoff for alcohol and drug dependence before engagement with BFO (OR${}_{\text{alcohol dependence}}$ = 0.39, *P* = 5.51 ×10^−9^, OR${}_{\text{drug dependence}}$ = 0.50, *P* = .005) were also significant. Pre-engagement anxiety was significantly associated with repeated misclassification on post-engagement anxiety and depression (OR${}_{\text{anxiety}}$ = 1.37, *P* = .021, OR${}_{\text{depression}}$ = 1.64, *P* = .0002). Repeated misclassification on post-engagement anxiety was significantly associated with female gender (OR = 1.33, *P* = .003), and misclassification on alcohol dependence was associated with participants undershooting the cutoff for depression pre-engagement (OR = 0.75, *P* = .029). Overall, we found no evidence of an association of prediction inaccuracy with ethnicity.

We have therefore identified that it is possible to construct predictive models of the DI effectiveness from initial first-contact symptom profile using retrospective analysis of historical data. Predictive performance was reduced for certain user groups suggesting user heterogeneity in response to the DI and in line with previous analyses linking the associations of DI outcome with participant pre-engagement characteristics.

## Discussion

Our study was a retrospective exploration of real-world, user-reported data from a digital health intervention for SUD, and the first of its kind to use a heterogeneous and high-acuity sample. We used the example of BFO where 93% of users initially screened positive for dependence on a variety of substances, including Class A drugs such as crack, cocaine, and heroin. Seventy-two percent were dual diagnosis users who additionally screened positive for anxiety or depression.

Our analysis described user heterogeneity and identified factors implicated in user responses to this DI. Specifically, our analysis demonstrated how symptom improvements during BFO engagement compared with effects of belonging to a vulnerable patient group, such as women.^31^ Differential severity of a variety of symptoms reduced at a slower rate for women compared to men, suggesting an experience of SUD specific to women. Our findings agree with previous research investigating gender-specific responses to DIs for SUDs.^32–34^ This is of particular relevance given the relatively high proportion of women users for both BFO (43%, 47% in community environments) and other SUD focused DIs,^15^^,^^34^^,^^35^ relative to gender ratios in individuals presenting to SUD treatment,^30^ and the potential DIs therefore present for providing treatment for substance-using women. Supporting women with their specific challenges within gender-specific DI content, for example, on psychiatric comorbidities, trauma, and intimate partner relationships is therefore likely to be clinically beneficial.^36–38^ This approach aligns with one of the strategic objectives of the WHO Global strategy on digital health; to promote health equity and gender equality.^39^

User heterogeneity is also suggested by our observation that post-engagement symptom prediction based on first-contact symptom profile was more difficult for specific BFO user groups, for example, simultaneous alcohol and drug users, prisoners, and women. We speculate that this may be due to the possibility of compensating abstinence from one substance with use of the respective other substance (simultaneous alcohol and drug users), small user group size (prisoners), and different experiences of SUD and recovery from it (women).

Since our findings illustrate association rather than causation, the guidance they can provide about which users will benefit from BFO cannot be determined. As prediction accuracy for retention was low, that is, we are not able to predict whether at least a second use event will take place, it seems precipitate to offer the BFO program to select addiction service clients. Similarly, the implications of our findings for precision medicine are limited because we have examined sources of user heterogeneity between user groups rather than individuals. Instead, however, they illustrate sources of user heterogeneity which suggest changes in intervention design and delivery, for example, for users with specific challenges, such as women or users with dual diagnosis. They also illustrate possible levers DI providers can operate on to achieve DI success, such as integration of a DI with structured face-to-face support. The evidence we produced sufficiently motivates follow-up RCTs on the effect of DI user-stratified support systems with potential human elements.

The main limitation of our study is the conditionality of our findings about factors in DI outcomes on user retention, which was found to be low and may be improved by offering human support to users at risk of levels of engagement too limited to support behavior change. We identified only minimal baseline differences between users who were retained and those who dropped out after first use, but there may be differences on variables unmeasured, for example, BFO program acceptability or benefit. This limitation is shared by similar real-world studies, who encounter similar rates of early disengagement.^17^

Further, our definition of user engagement is not based on frequently used indicators of behavioral engagement found in log data, such as the frequency of use, time spent, and number of completions of DI modules.^15^^,^^17^ Instead, we define engagement as coming back at least once to complete a second BFO assessment. We believe that our definition is more tolerant of individual preferences of engagement intensity,^12^^,^^14^ for which evidence is emerging. Our definition also aligns with recent conceptualizations of engagement which highlights affective and cognitive components of engagement beyond the traditionally measured behavioral investment.^40^^,^^41^

## Conclusion

As the deployment of DIs continues to proliferate, and users of these interventions support the generation of complex datasets, it becomes incumbent on DI developers to ensure that the data they collect from users are utilized in innovative ways, thus optimizing clinical benefits. This study uses basic service interaction data which may be available to researchers at a minimum, focusing on user heterogeneity driven by specific static user characteristics. Developers and commissioners of digital health interventions may adapt this analysis to better understand outcomes and the way these are impacted by user heterogeneity in order to evolve their products to deliver more effective services.

## Supplementary Material

ooad072_Supplementary_Data

## Data Availability

Data reported in this article cannot be shared publicly—this is due to restrictions placed on the sharing of personal health data by the UK General Data Protection Regulations (GDPR). However, requests to share data may be considered when appropriate ethical and data privacy and security standards have been met—requests for sharing of data should be sent to SE (sarah.elisondavies@lifeworks.com).
