# Supplementary material for: Identifying factors associated with user retention and outcomes of a digital intervention for substance use disorder: a retrospective analysis of real-world data
Source: JAMIA Open. 2023 Sep 2;6(3):ooad072. doi: 10.1093/jamiaopen/ooad072 (PMC10474970; doi:10.1093/jamiaopen/ooad072)
Supplement: ooad072_Supplementary_Data [file ooad072_supplementary_data.pdf]

Supplementary Material

Clinical model of BFO

BFO content was developed by researchers and clinicians in the field of SUD and behavior change, in consultation with multiple stakeholders, including people accessing SUD treatment and people in long-term recovery from SUD, practitioners, service managers and commissioners.[18, 19] BFO is endorsed by the UK National Institute for Health and Care Excellence (NICE) and the content of the program complies with NICE guidance around treatment for SUD.

Access to BFO is provided after account creation and completion of an assessment battery of the user's demographic characteristics, substance use, substance dependence, mental health, quality of life, biopsychosocial functioning and treatment goals. Account creation requires an access code from an SUD treatment service commissioning BFO, and an email address. Service staff are trained to assist during the entire first contact with BFO and beyond. Additionally, the BFO website features an informational video about BFO, accessible without an account, for individuals who have not accessed treatment services yet and hence have not been referred to the program by service staff. Users can access BFO on personally owned devices and devices provided at the service.

BFO is integrated with face-to-face interventions at commissioning SUD treatment services. Practitioners and clinicians are trained via an e-learning platform to interlock clinical practice with BFO by referring to and reflecting on learnings, and offering support. Another source of support and motivation available to users at some treatment services are peer mentors who have experience of using BFO and are trained to support others to engage with the program. The BFO version used in correctional services is delivered as part of a manualized, 8-session structured group or one-to-one intervention that is delivered by trained staff. It is to date the only DI approved by Her Majesty's Prison and Probation Service as an Effective Regime Intervention, and accredited by the UK Ministry of Justice Correctional Services Advice and Accreditation Panel.

BFO builds on the six-domain Lifestyle Balance Model [18] which details domains of functioning implicated in substance use disorder: "impact of emotions", "unhelpful behaviours", "physical

sensations”, “difficult situations”, “negative thoughts”, and “lifestyle”. BFO visualizes a user’s degree of functioning in each of these domains based on their answers from the assessment battery included in the program. After the initial pre-engagement assessment, these answers must be updated at least bi-weekly in order for users to be able to continue accessing the clinical content in BFO. Users who have updated their assessment at least once are considered retained in treatment, and otherwise, dropouts.

Each domain is associated with slide series based psychoeducation on the impact of this domain on functioning (“Information Strategy”), and an interactive, skills building exercise (“Action Strategy”). Domain modules can be accessed in any desired order and pace. The interactive exercises make use of a range of evidence-based behavioral change techniques, including refusal and assertiveness skills, emotional regulation, coping strategy enhancement, mindfulness-based cognitive therapy, motivational enhancement, cognitive restructuring, reward and reinforcement, harm reduction and crisis management.[19] All BFO pages are supported by audio or video content. Learnings from psychoeducation and interactive exercises, including user input, can be downloaded. They can also be sent per mail to the user and their BFO recovery supporters which can be nominated in the program through entering up to three email addresses.

The mobile Companion app complements the BFO web app. Specifically, it makes use of geolocation technology to provide alerts of user-inputted locations which bear individual risk of substance use, and calendar and time alerts for planned activities of achievement and enjoyment, and planned steps towards a life goal.

Details of random forest prediction

Pre-engagement answers on items associated with the SDS are used as predictors only if post-engagement answers on these items are predicted. 500 trees were grown in an individual random forest. The number of predictors randomly sampled at each node split corresponded to the square root of the number of predictors included to predict an outcome. Random forest performance was assessed by calculating ROC curves and areas under them which were averaged across folds. Accumulated local effects, used to illustrate the effects of drivers of prediction on post-engagement anxiety, describe the main effect of an answer on one of these drivers on the predicted probability of anxiety compared to the average prediction.

Supplementary Figures

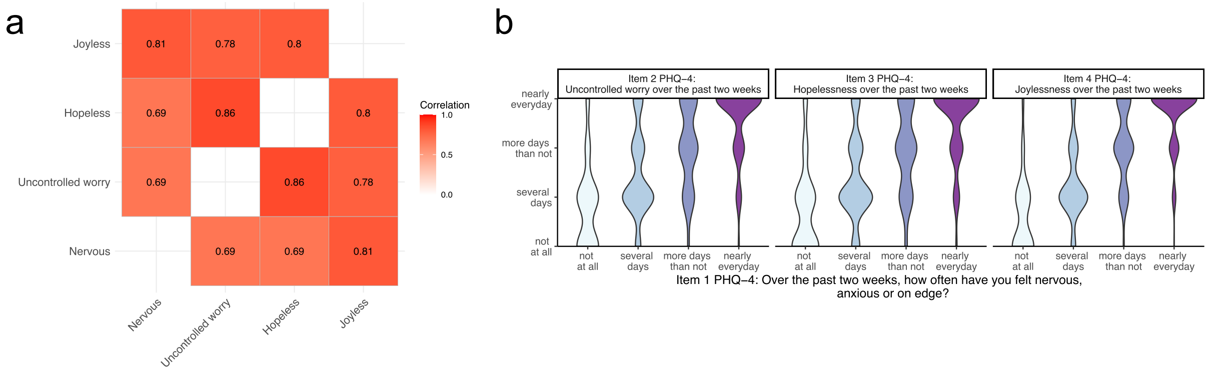

Figure S1: **Correlations between PHQ-4 items.** Correlation matrices as in (a) are estimated alongside other parameters of our regression model inspired by [20]. This allows accounting for the high correlation between items of a scale (range ρ for PHQ-4 items: 0.69 - 0.86) when modelling single items. High correlation is also suggested by a high rate of co-occurrence of the same answer category on two items of the same scale in the data, as shown for the PHQ-4 in (b).

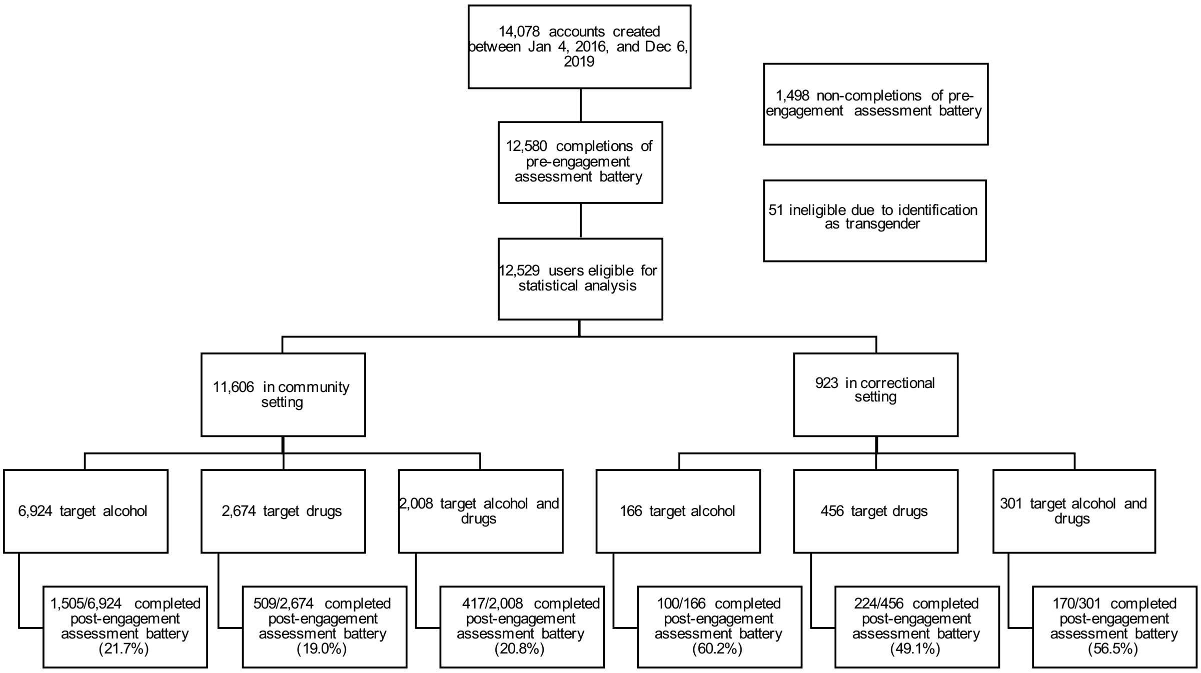

Figure S2: **Participant flow through BFO and eligibility.** Completion of the pre-engagement assessment battery is defined as providing an answer for at least one item per psychometric (sub-) scale. Information about missing data in the dataset used for statistical analysis is provided in Table 2.

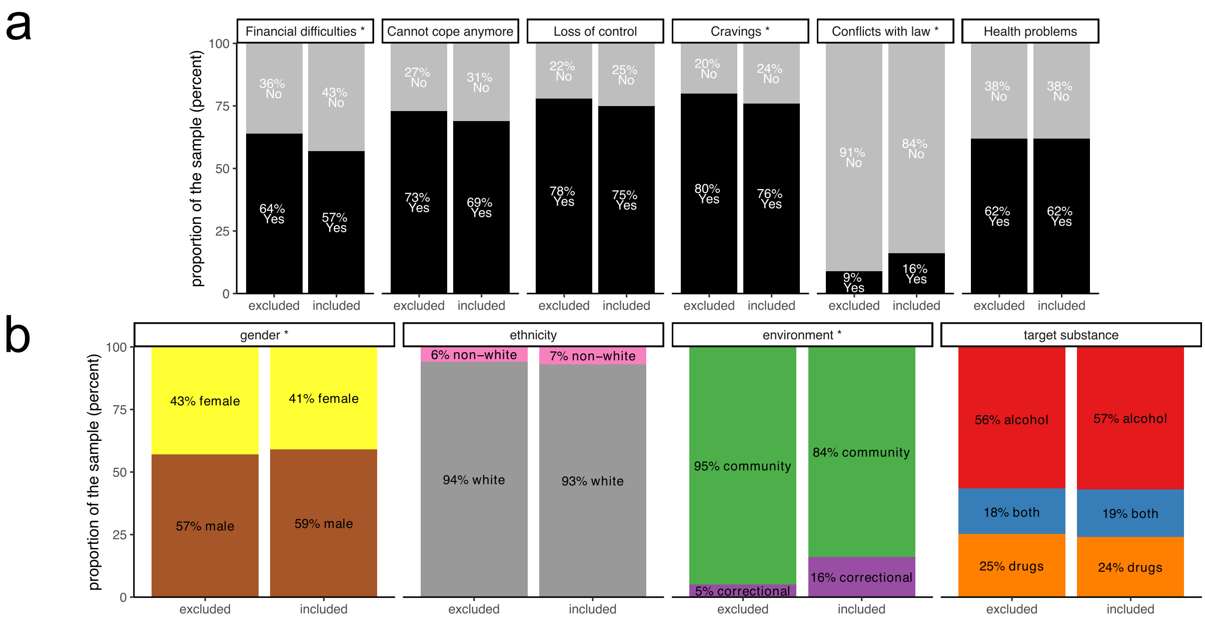

Figure S3: **Differences between participants in- and excluded on basis of availability of a post-engagement assessment.** Stacked bar plots showing differences in responses between participants with and without post-engagement assessment data for (a) a selection of questionnaire items and (b) sociodemographic characteristics. Each color block reflects a different categorical level for the questionnaire item displayed. An asterisk indicates statistical significance of the difference between participants with and without post-engagement assessment data, suggested by regression models.

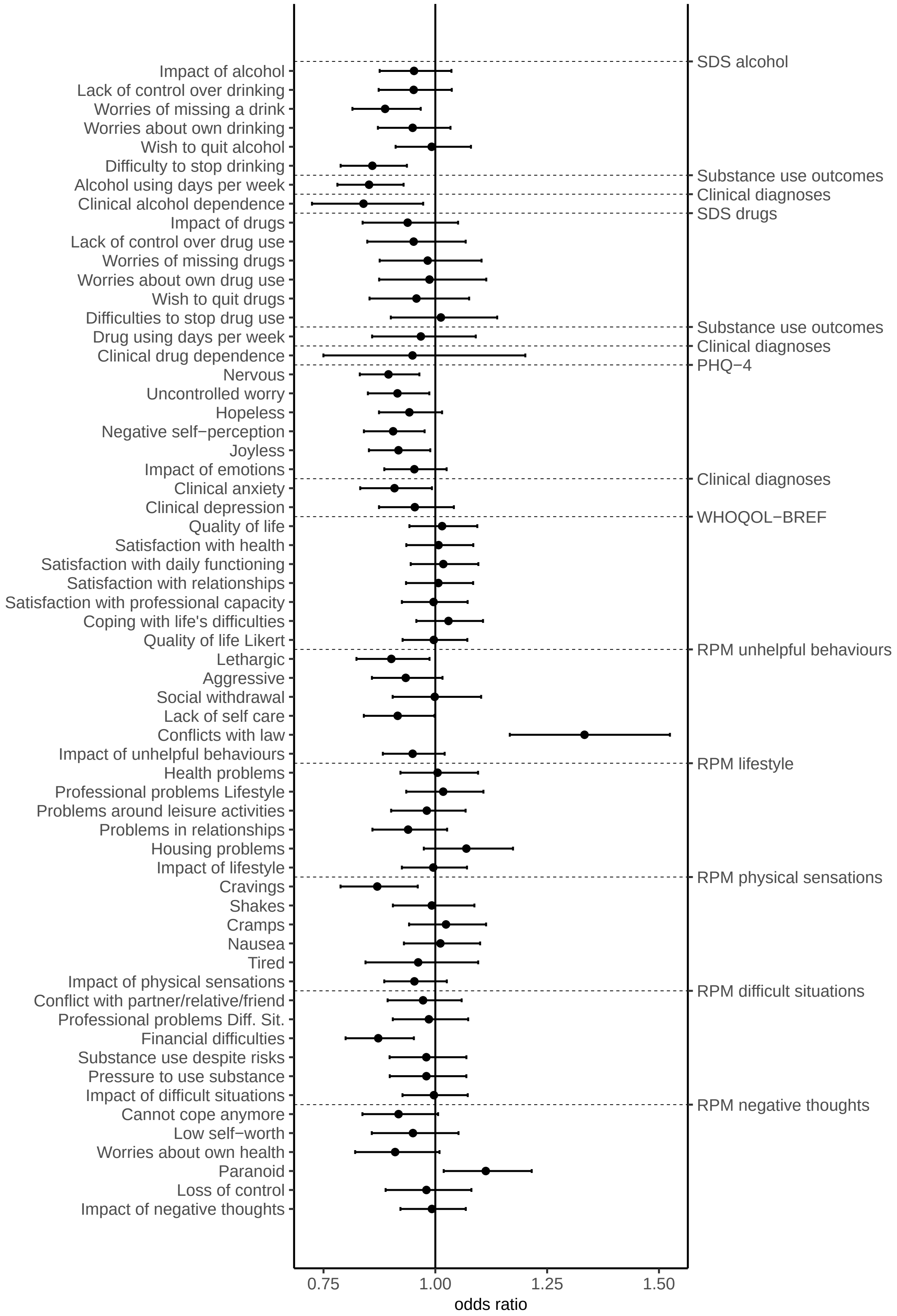

Figure S4: **Association of participant pre-engagement clinical complexity measured on item and aggregate levels with retention.** Data are odds ratios of occupying higher

categories on items or fulfilling diagnostic core criteria for a specific psychiatric disorder by participants with a post-engagement assessment vs. those without. They are shown with their 95% confidence intervals, the severity of dependence scales, the PHQ-4 and the WHOQOL-BREF with affiliated items.

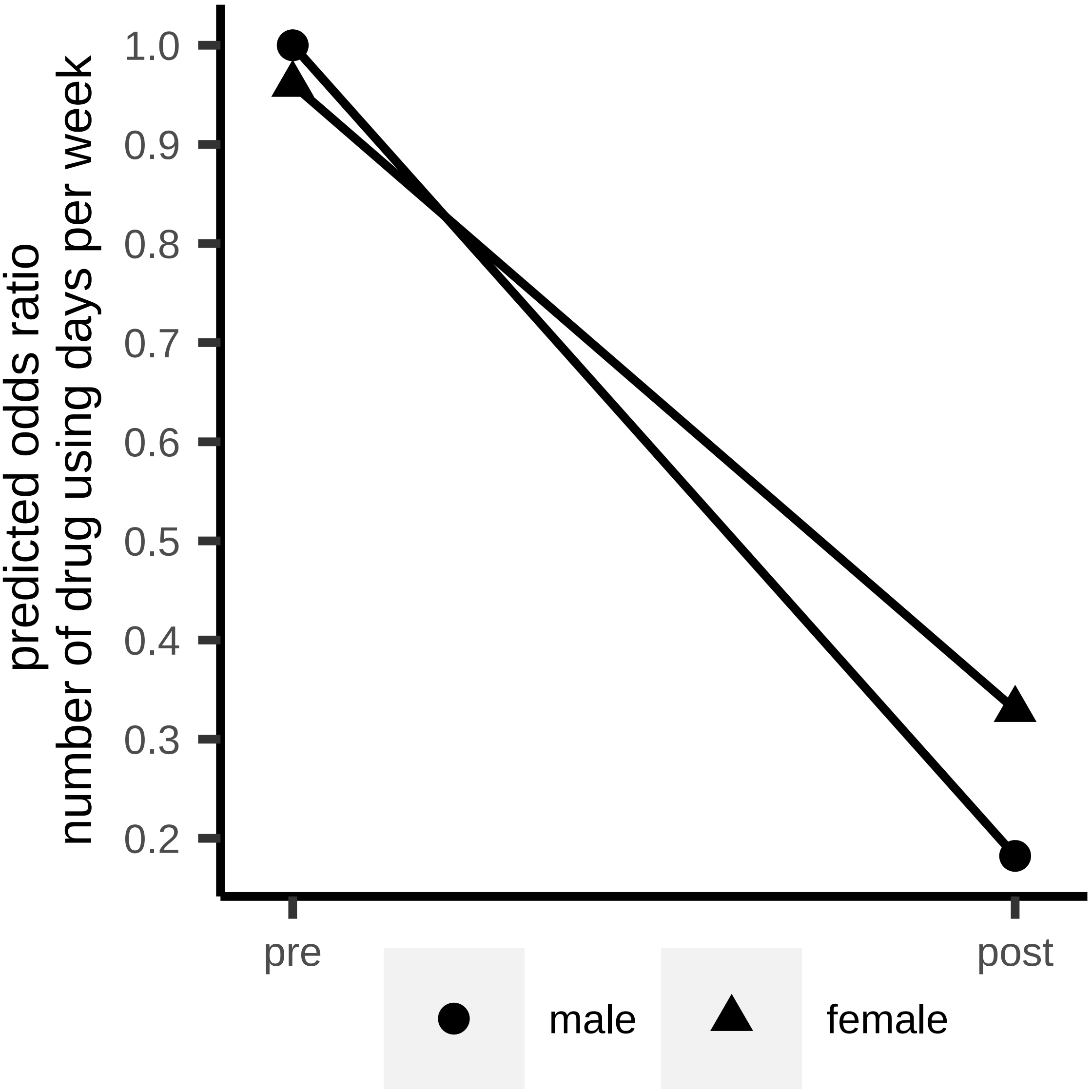

Figure S5: **Effect of the interaction of time point and gender on drug using days per week.** Shown are predicted differences in odds of using drugs on more rather than less days during a typical week. The reference group is men participants at baseline (OR = 1).

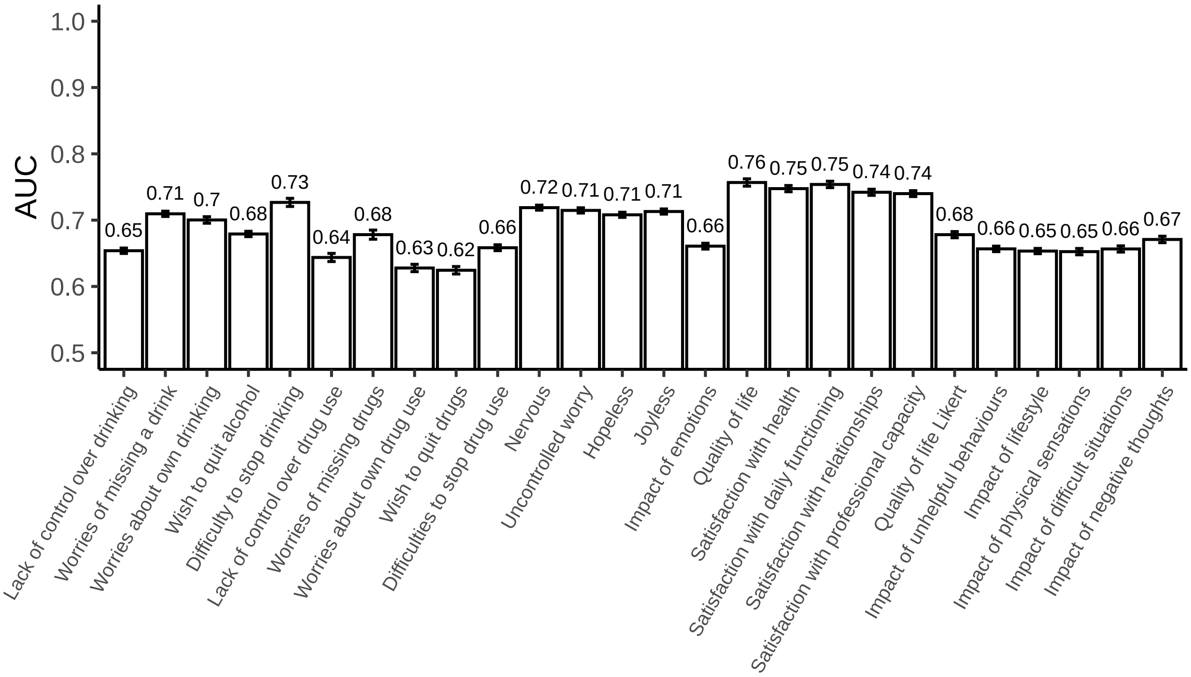

Figure S6: **Areas under the curves for ordinal outcomes.** Bars show the averaged prognostic performance of random forests in 100 validation datasets with a 95% confidence interval.

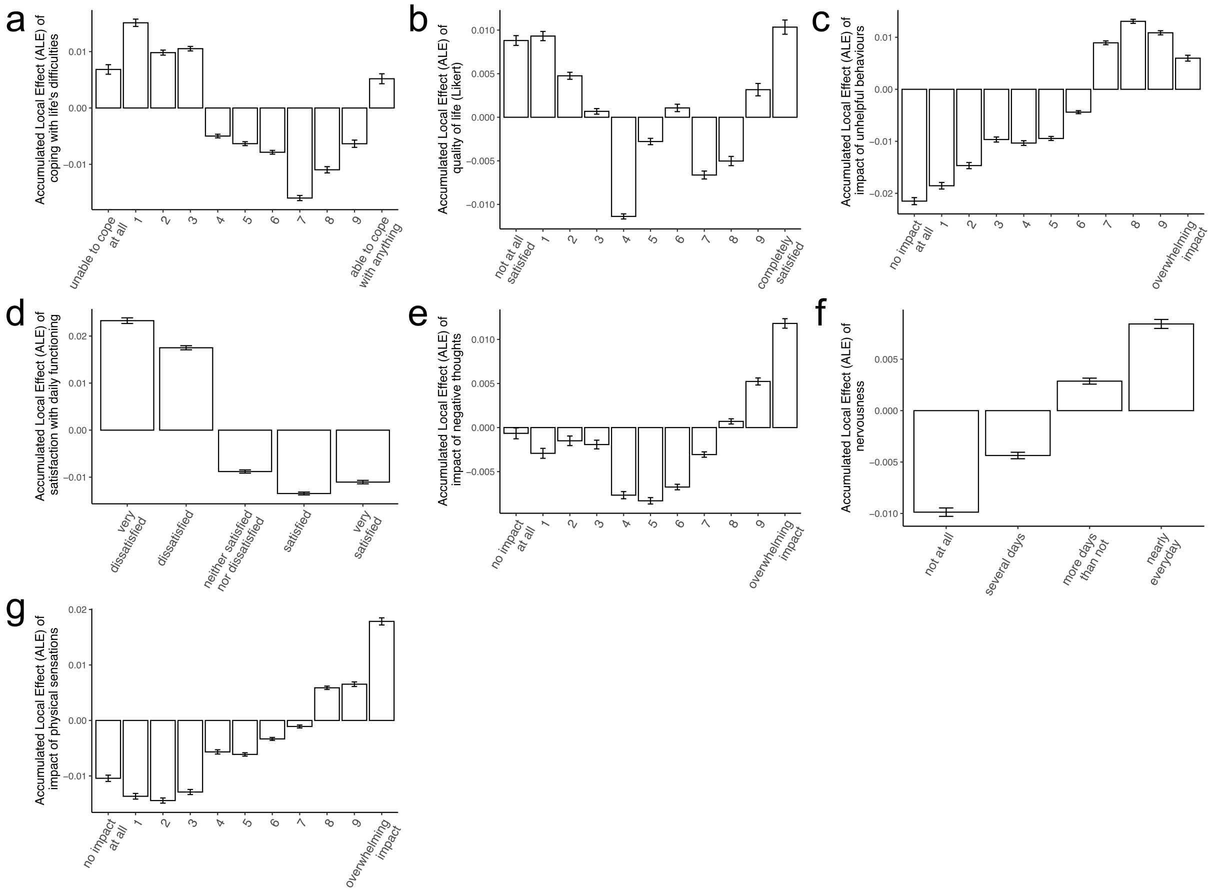

Figure S7: **Accumulated Local Effects of seven features on the prediction of anxiety.** Shown are features ranking 4th to 10th based on aggregated importance across functioning related outcomes.
